# Telemedicine and Telehealth in Urology: Uptake, Impact and Barriers to Clinical Adoption

**DOI:** 10.3389/fsurg.2022.911206

**Published:** 2022-06-30

**Authors:** Nithesh Naik, Shreyas Raghavan Nandyal, Sanjana Ganesh Nayak, Milap Shah, Sufyan Ibrahim, B. M. Zeeshan Hameed, Ananth Patil, Gopika Suresh, Pritam A. Shetty, Bhavan Prasad Rai, Rajeev TP, Patrick Rice, Suraj Jayadeva Reddy, Nandakishore Bhat, Deepesh Garg, Piotr Chlosta, Bhaskar K. Somani

**Affiliations:** ^1^ Department of Mechanical and Industrial Engineering, Manipal Institute of Technology, Manipal Academy of Higher Education, Manipal, Karnataka, India; ^2^ iTRUE (International Training and Research in Uro-oncology and Endourology) Group, Manipal, Karnataka, India; ^3^ Gandhi Medical College, Kaloji Narayana Rao University of Health Sciences, Secunderabad, Telangana, India; ^4^ Department of Computer Science and Engineering, Manipal Institute of Technology, Manipal Academy of Higher Education, Manipal, Karnataka, India; ^5^ Robotics and Urooncology, Max Hospital and Max Institute of Cancer Care, New Delhi, India; ^6^ Kasturba Medical College, Manipal Academy of Higher Education, Manipal, Karnataka, India; ^7^ Department of Urology, Father Muller Medical College, Mangalore, Karnataka, India; ^8^ A.J. Institute of Medical Sciences and Research Centre, Mangalore, Karnataka, India; ^9^ Department of Urology, Freeman Hospital, Newcastle upon Tyne, United Kingdom; ^10^ Department of Urology, Government Medical College Hospital, Guwahati, India; ^11^ Department of Urology, University Hospital Southampton NHS Trust, Southampton, United Kingdom; ^12^ Department of Urology, Kasturba Medical College, Manipal Academy of Higher Education, Manipal, Karnataka, India; ^13^ Department of Information and Communication Technology, Manipal Institute of Technology, Manipal Academy of Higher Education, Manipal, Karnataka, India; ^14^ Department of Urology, Jagiellonian University in Krakow, Kraków, Poland

**Keywords:** telemedicine, urology, telehealth, COVID-19, patient satisfaction

## Abstract

Telemedicine has great potential in urology as a strong medium for providing patients with continuous high-quality urological care despite the hurdles involved in its implementation. Both clinicians and patients are crucial factors in determining the success of tele-consults in terms of simplicity of use and overall satisfaction. For it to be successfully incorporated into routine urological practice, rigorous training and evidence-based recommendations are lacking. If these issues are addressed, they can provide a significant impetus for future tele-consults in urology and their successful deployment, even beyond the pandemic, to assure safer and more environment-friendly patient management.

## Introduction

The COVID-19 pandemic has nudged more surgeons and practitioners into inculcating telemedicine into their routine practice. Although used interchangeably, telehealth may strictly differ from telemedicine in that it encompasses a larger range of remote healthcare services than telemedicine. In addition to clinical services, telehealth can refer to remote non-clinical services such as provider training, administrative meetings, and continuing medical education. While there are still certain challenges in incorporating telemedicine into mainstream surgical practice, we wanted to explore the extent to which clinicians safely and effectively incorporate telemedicine. Also, the aspects of patient care that are best addressed *via* telemedicine and how this transition affects physician-patient relationships and issues such as its impact on climate and cost.

Telemedicine offers the advantage of being a practical, alternate solution, especially during times of pandemic, to significantly reduce the transmissibility of communicable disease, ensuring better protection for physicians and patients (1). According to a study from Borchert et al., urologists followed a triage mechanism concerning the implementation of telemedicine to both COVID-19 and non-COVID-19 patients, emphasising that consultations can be managed in a patient and physician safety-conscious manner, reducing transmissibility while ensuring satisfactory consults (2). Some urologists witnessed voluntary cancellation of in-person visits by patients themselves who opted for tele-consults (1). This emphasises the fact that telehealth has gained favourability among patients who are anxious about disease transmission, especially in the setting of the current pandemic.

A literature search was done for English language articles over the last two decades from 2020 to 2021, and present in Scopus, Pubmed, MEDLINE, Clinicaltrials.gov, Web of Science (WoS), and Google Scholar. A total of 38 articles were identified on the initial search. After screening, 17 articles were identified as related to uptake, impact, and barriers among the clinicians for the adoption of telemedicine in urology. The search was conducted by using a combination of the following terms: “telemedicine”, “clinicians”, “urology”, “telehealth”, “prostatic neoplasms”, “urinary bladder neoplasms”, “kidney neoplasms”, “testicular neoplasms”, “prostatic hyperplasia”, “urinary calculi”, “sexual dysfunction”, “physiological”, “erectile dysfunction”, “infertility”, “urinary tract infections”, “urinary incontinence”, “prostate cancer”, “bladder cancer”, “kidney cancer”, “testis cancer”, “benign prostatic hyperplasia”, “urinary stone”, “sexual dysfunction”, “erectile dysfunction”, “infertility”, and “genitourinary trauma”.

**Figure 1:**
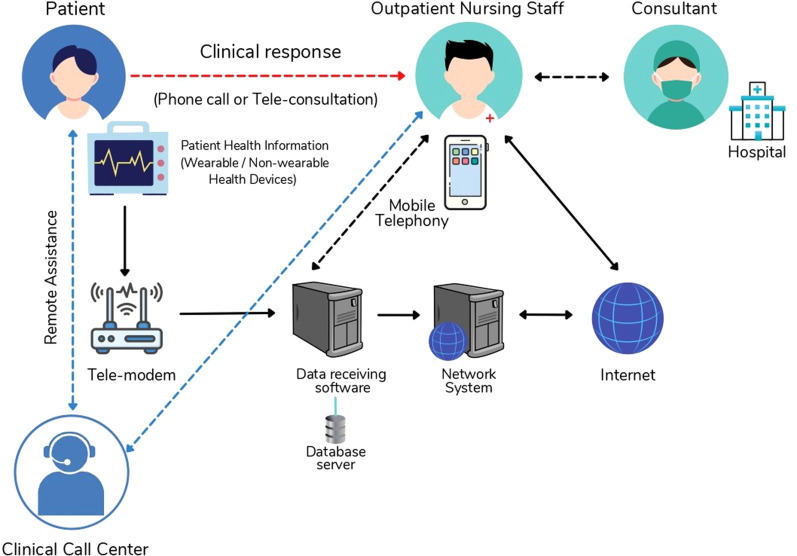
Network architecture of telehealth consultation.

Telemedicine has also offered the chance to address sexual health-related urologic problems, in a manner that is comfortable to the patients (3). Urological cancer care and counseling were as effective as in-person visits in one study (4). Another major advantage of telemedicine compared to routine clinic visits was the reduction in carbon footprint attributed to such a transition, emphasising the fact that telemedicine can help contribute to a more environment-friendly clinical practice (5, 6). Figure 1 shows the network architecture to establish a connection between patient and consultant using a telehealth application.

Physicians are keen to incorporate telemedicine into routine urology practise, but there is still a need for more active support and active guidelines with formal training (7, 8). Barriers to accessing appropriate and adequate technology remain an important concern that needs to be addressed (9). Patients who were not English proficient and those that came from areas with an unstable Internet connection, were less likely to use telemedicine urology consults (3). Similarly, patients were keen at the outset to take up telemedicine due to an unclear understanding of indications and concerns related to reimbursement (7, 9). Among the users that adopted telemedicine, however, there was a significant degree of satisfaction with health information received *via* this medium.

Data security and breaches in confidential health information are key issues that were identified and need vigilance and improvements (3). Additionally, there might be some inaccuracies and loss of crucial clinical information during transmission of information *via* telephone consults (1). While there are challenges in implementation, there was a good response in adapting telemedicine into clinical practice. A cross-sectional study found that since the start of the pandemic, urologists' implementation of telemedicine has increased significantly, with one survey suggesting that it nearly tripled compared to pre-pandemic times (9). This exemplifies the need and urgency with which such practices were incorporated and might be a pointer to a similar response in future pandemic and non-pandemic times.

Through this review, we try to address the factors affecting the uptake of telemedicine and telehealth in routine urological practise, the impact it has had on the delivery of patient care, and the several challenges and concerns faced by urologists in its adoption.

## Role in Preventing Transmission of Communicable Diseases

Before COVID-19, the role of telehealth consultations was minimal in routine urological practice. Several studies have therefore been undertaken to evaluate the suitability of incorporating telehealth in urology. A global cross-sectional study done during the pandemic tried to address urologists' perceptions of the utility of in-person clinic appointments, the feasibility of substituting traditional care with telemedicine, and the current barriers to its implementation. It found that close to 80% (*n* = 244) of urologists were keen on continuing the use of tele-consults in their practice after the pandemic (9). The study also found that globally telemedicine in urology tripled, compared to the pre-pandemic times. Urologists however identified three major barriers in the implementation of tele-consults, patients' inability to understand and effectively utilize this technology, lack of access to the required technology, and reimbursement concerns.

Borchert et al. found that adopting a triage system with respect to the risk, stratifying patients in urgent need of urological care, COVID-19 positive and COVID-19 negative patients, could show promise in safely caring for patients, whilst preventing communicable disease transmission (2). Team members from urology care did not contract the virus and could safely attend to patients at the same time while employing this strategy.

A study from Northern Italy concurred with the same observation, where Luciani et al. found that a “targeted virtual approach” would prove to be of greater benefit over the cancellation, especially in patients with advanced urological malignancies (1). The study also identified that inaccuracies might creep in during tele-consults and crucial data might be missed out on, and recommended that it was imperative to maintain robust electric health records and services from expert staff members to tackle this issue. Table 1 summarizes some of the key focus, findings of the studies discussed, and perspectives of urologists towards acceptance and implementation of telemedicine in their clinical and surgical practices.

**Table 1:** Summary of recent studies in urologists’ perspectives on telemedicine.

Study	Number of consults and type of study	A key focus of the study	Impact of Tele-urology	Barriers to implementation
Luciani et al. (1)	928 Retrospective Study	Role in preventing transmission of communicable diseases	Effective substitution of at least part of traditional urology face to face consults with telemedicine can be possible	Inaccuracies in data interpretation and data security concerns
Borchert A et al. (2)	53 Prospective Study	Following a triage mechanism with the implementation of telemedicine to reduce the risk of transmission between patient and provider	Out of 34% of consults that were COVID 19 positive, 44% were successful *via* Telemedicine alone. No team member contracted the infection during this study	An effective triage system for patients with contagious diseases can promote optimization of care and prevent patient and physician health hazards, *via* transmission of communicable diseases.
Rabinowitz et al. (3)	1,949 Cross-Sectional Study	Sexual Medicine	Improved compatibility of telemedicine with sexual medicine for safe and private discussions between patient and clinician	Data protection and confidentiality concerns
Margolin et al. (4)	115 Cross-Sectional Study	Sexual medicine and other sensitive issues related to cancer care	Great patient satisfaction with tele-consult	Routine technological barriers with regards to connection and ability to use the platform
Filfilan A et al. (5)	80 Prospective Cohort	Carbon Foot Print and Cost Reduction	Environmental friendly practise can be promoted if telehealth is implemented judiciously	Judged by a majority as good teleconsultation and rated as a good experience
Croghan S et al. (6)	1,016 Prospective Study	Carbon Foot Print and Cost Reduction	Improved environmental outcomes with uncompromised urologic care	Enhanced efficiency over multiple domains as an alternate for physical attendance
Connor et al. (8)	144 Cross-Sectional Study	Support From Organizations and Need for Telemedicine Guidelines	General satisfaction was expressed with utilizing telehealth in urology	Lack of formal training and absence of established guidelines
Beller et al. (10)	20 Prospective Outcomes Study	Comparing traditional cystoscopy with nurse-led tele-cystoscopy	Accessibility of care for patients in remote areas can be increased	- NA -
Lambracos et al. (11)	Retrospective audit	Carbon Foot Print Reduction	Increased service efficiency and environment-friendly surgical practice	Optimise service efficiency with environmental-friendly alternative
Andino et al. (12)	600 Retrospective Study	Procedure Related	Video visits can cater to almost all of the patient's concerns and can serve as an effective substitute for inpatient visits	The demographic barrier is age. Older people are not as likely as younger people to readily take up video visits
Chesnel et al. (13)	358 Cross-Sectional Study	Sexual Medicine and Other Sensitive Issues Related To Cancer Care	High satisfaction among patients and efficient alternative for physicians	Cognitive impairment of patients and inability to participate effectively in virtual care, inadequate/ flawed data gathering and data interpretation, and lack of traditional physical examination as a component of complete care of the patient.
Hughes et al. (14)	290 Prospective Outcomes Audit	Procedure Related	Reduced costs and improved efficiency with virtual clinics	Wider uptake and optimised use of healthcare resources
Nourian et al. A (15)	2,008 Retrospective Study	Procedure Related	Telehealth alone could provide comprehensive clinical care for some diseases	Many patients were eventually seen face-to-face for urological examination and procedures
Balzarro et al. (16)	420 Prospective Comparative Study	Telephone follow-up was able to detect organ prolapse recurrence and other symptoms*;* Increased patient satisfaction with the virtual clinic was reported.	A feasible and reliable tool to detect POP	Misinterpretation of incontinence symptoms might lead to misclassification
Wadensten et al. (17)	123 Randomized Controlled Trial	Procedure Related	Improved incontinence related outcomes in women—App-based service provides a decent alternative to pharmacological treatment or other conservative management, thus increasing access to care	Access and literacy to advanced eHealth services
Ong et al. (18)	465 Plan-Do-Study-Act method	Procedure Related	Implementation of telemedicine service for ureteric colic patients adequately reduced the need for in-person visits and time to review patient details with minimal capital expenditure while maintaining patient safety	Limited generalizability of findings

## Carbon Footprint, Time and Cost Reduction

A reduction in inpatient time and cost as well as significant drops in CO_2_ emissions can be an impetus to adopting a more environmentally friendly practice. The impact of telehealth on reducing greenhouse gas emissions was studied and it was found that adopting tele-consults reduced transport to the tune of 52 miles per patient. This study alone saw a net decrease in CO_2_ emission by 1.1 tonnes (1). Croghan et al. in their study estimated that adopting tele-consults saved 1,257.8 h of patient travel time and a 6-tonne reduction in carbon emissions over 3 months (6). The study also evaluated the utility of virtual clinics across several other domains in terms of effectiveness and found that offering telehealth options to patients might prove just as valuable as in-person visits.

Beller et al., in their observational retrospective study of the National Hospital Response Framework Alert, found that virtual phone consults increased by 274% while saving each patient an estimated 22.7 km of travel on average. The study points toward how service efficiency can be optimised while providing an environmentally friendly alternative for routine clinical practice (10). Prospective audit of virtual clinic visits of patients was done over 6 years and found that virtual consults reduced the cost per clinic appointment by 93%, equating to a total saving of £12,006 during the study (11). This study also found that nurse-led virtual clinics provided a safe follow-up and also allowed to substantially reduce the cost of treatment by meticulously selecting between virtual discharge or an in-patient face-to-face visit, on a case-to-case basis (11).

The ureteric colic assessment was simplified by adapting tele-consults. Andino et al., in their study from a specialty center in Singapore, used telemedicine workflow as a quality improvement study using the PDSA method. They showed that ureteric colic telemedicine service reduced the number of face-to-face consultations and time to review patient data, without compromising patient safety. The wait time for patients was considerably reduced by adopting this approach. The study also found that patients saved on the cost and time spent on travel and follow-up clinic visits. At the same time, we're able to get earlier reviews at an average of 30.3 days, a significant improvement from the previous 60 to 90 days. This study also received great patient satisfaction (12).

## Sexual Medicine and Other Sensitive Issues

Telemedicine in sexual health-related urologic concerns received positive appreciation from patients and urologists alike. There was a steady increase in the number of male sexual health-related consults during the COVID-19 pandemic. Telemedicine helps create a comfort zone for the clinician and patient to discuss sensitive topics, such as impotence and infertility. Croghan et al. in their study of 115 patients, found that 77% of patients and 70% of physicians reported being “extremely satisfied” with most of the components of telemedicine encounters including communication, the scope for shared decision making, time devoted to patient concerns and overall effectiveness and convenience (6). Almost 78% of patients and 85% of physicians “strongly agreed” that they were able to comprehensively discuss sensitive topics about cancer care, uncompromisingly as in an in-person visit (6). This study also identified technological barriers to be the key limiting factor to the successful implementation of such encounters.

Rabinowitz et al. (3) tried to establish patient satisfaction in tele-urology consults in the domain of neuro-urology in 358 patients, and found that the mean efficiency of the telephone consultation was 9.3/10 (±1.5) (3). The mean global satisfaction was 9.0/10 (±1.3). However, this study found that a sizeable number preferred an in-person consult is available to them. This study found that cognitive impairment, difficulty to obtain key patient details, and lack of the traditional physical examination were unfavorable to the efficiency of teleconsultation.

## Guidelines for Successful Implementation

A German study from Chesnel et al. tried to ascertain the suitability and comfort of telemedicine consults among urologists and patients (13). This study found that patients largely favoured telemedicine consults and that users were more likely to implement telemedicine after the COVID-19 pandemic. However, the usage among German urologists remains low and they feel that there needs to be active support in the form of Societal guidelines for better implementation. Among patients that were offered tele-consults, nonusers were concerned with unclear indications for telemedicine and lesser reimbursements during telemedicine than in-person visitations.

Rodler et al. identified training in telemedicine among urologists as a key determinant of the success of tele-consults. Experience with telemedicine was assessed in 2 categories: technical determinants and patient communication. Variables were rated using a 5-point Likert Scale. Providers were more concerned about lack of adequate training in billing, rather than deficits in communication and equipment use (7). 87% of providers felt at ease while discussing sensitive topics using telehealth, while only 55% felt comfortable using telehealth (*p* < 0.001). Therefore, this study identifies that the providers would benefit from focused training in telemedicine for a successful implementation.

## Procedure-Related Concerns

Connor et al. conducted a study to evaluate the feasibility of using nurse-led cystoscopy services and virtual patient consults *via* telemedicine, where urologic advanced practitioners performed cystoscopies that were interpreted virtually by urologists. This study found that such a model can improve access to urology consults for patients in remote locations and may lead to greater acceptance of nurse-led procedures (8).

Lambracos et al. did a study to determine the feasibility, reliability, and overall patient satisfaction of telephonic follow-up in women treated for stress urinary incontinence and pelvic organ prolapse. The study found that there might be a misinterpretation of initial symptoms and arriving at these diagnoses to start with, but the overall follow-up was feasible and reliable in women not reporting incontinence. This highlights a limitation of such tele-consults while dealing with incontinence-related problems (11).

Hughes et al. (14) in their study established the feasibility of video visits by patients as opposed to in-person visits and used the metric of “revisit” rates to determine success. They found that revisit rates were more when the initial visit was in an in-patient setting as compared to a virtual clinic approach. This can be attributed to previously scheduled in-patient appointments. Revisit rates due to medical concerns were similar across telehealth and in-person visits.

Patients with urological benign diseases whose clinic visits had been cancelled due to the COVID-19 pandemic were evaluated by Checcucci et al. (19) using telemedicine with phone-call visits as a feasible method for follow-up care. A telephone call was made to 607 of the individuals on the list. Many instances (531/607) revealed no need for in-person or emergency appointments since the symptoms were stable. 81.5 percent of patients (495/607) were more concerned about the possibility of infection than their urological disease. When it came to patient satisfaction with the utility of phone visits (rated at 4/5), the median score was 5/5 for the comprehensibility as well as ease of communication of exams, while only 53% of interviewees had access to the basic tools needed to conduct a real telemedicine consultation in accordance with the guidelines. For infectious disorders like COVID-19, the use of telemedicine reduces the frequency of needless visits to healthcare facilities and is a significant strategy in limiting the risk of transmission. A digitalization process must be pursued by infrastructures, health care providers, and patients in order to make televisit more widely available.

The clinical pathways for urology patients during the COVID-19 pandemic were discussed in a study by Simonato et al. (20). Since the outbreak of the COVID-19 pandemic, several urology centres have had to reorganise their clinical operations due to the massive reallocation of health resources. Preoperative, intraoperative, and postoperative care pathways have been established by an Italian group of urologists for the treatment of COVID-19 pandemic patients undergoing urgent urological surgeries or non-deferrable oncological interventions in Italy. In order to reduce the number of hospitalizations and thus the risk of infection, the diagnostic and staging process must be simplified. The recommendation suggested is that an accurate triage for COVID-19 symptoms be undertaken by telephone both before and during hospitalisation, as nasopharyngeal swabs are not mandated by tight laws. To make it easier for patients to return home and remain there after their hospital stay, we recommend that as many instructions as possible be given to them. Patients should be discharged from the hospital in a stable and healthy state in order to reduce the likelihood of a return visit. Post-discharge checks should be scaled back or rescheduled, and a reliable communication mechanism for telemonitoring patients should be put in place.

## Challenges of Telemedicine and Future Perspectives

Telemedicine holds great promise in changing the way urology consults are carried out. There seems to be time and cost reduction, with satisfactory patient and clinician outcomes, suggesting that adopting such a practice holds a great scope in a future way beyond the pandemic. Carbon emission reduction and a greener surgical practice are added advantages of adopting such a practice. On the same token there exist challenges and barriers in the form of miscommunication, data privacy, security concerns, and lack of adequate training and guidelines, all of which needs to be addressed to ensure the success of such novel clinical practice (9).

In low-resource settings, in particular, wherein individuals lack sufficient diagnostic opportunities, have limited access to medical services due to financial conditions, a general lack of interest in healthcare, and their incapacity to get to the hospital on their own—particularly in the case of seniors living alone—along with a dire need for preventative measures and ongoing medical care in this group, telemedicine-based services has been a welcome change to address the constraints.

There is a paucity of literature concerning the use of telemedicine before the COVID-19 pandemic, which is a major limitation of his study. There is also inadequacy stemming from a lack of long-term follow-up in patients who were shifted to receive telehealth consultations and would require longitudinal studies to better study the potential benefits of telemedicine.

## Conclusion

The review identifies that telemedicine in urology holds promise as a powerful medium for the delivery of uninterrupted high-quality urological care to patients. Patients play an important role as a determinant in the success of tele-consults with regards to ease of use and ultimate patient satisfaction. The primary limiting factors for the effective implementation among urologists lie in the fact that there is inadequate formal training and evidence-based guidelines to support its successful implementation. These concerns if addressed can prove to have a great impetus for future tele-consults in urology and its effective deployment, even after the pandemic, to ensure safer and more environment-friendly clinical and surgical practices.
